# Critical Parameters for the Development of Novel Therapies for Severe and Resistant Infections—A Case Study on CAL02, a Non-Traditional Broad-Spectrum Anti-Virulence Drug

**DOI:** 10.3390/antibiotics9020094

**Published:** 2020-02-21

**Authors:** Samareh Azeredo da Silveira, Andrew F. Shorr

**Affiliations:** 1 Combioxin SA, 8 rue de la Rôtisserie, 1204 Geneva, Switzerland; 2 Pulmonary and Critical Care Medicine, Medstar Washington Hospital Center, Washington, DC 20010, USA; andrew.shorr@gmail.com

**Keywords:** severe infections, pneumonia, virulence, broad-spectrum, nontraditional, toxins, endpoint

## Abstract

Background: Poor outcomes in severe and resistant infections, together with the economic struggles of companies active in the field of anti-infective development, call for new solutions and front runners with novel approaches. Among “non-traditional” approaches, blocking virulence could be a game changer. Objectives: This review offers a perspective on parameters that have determined the development path of CAL02, a novel anti-virulence agent, with a view to steering clear of the obstacles and limitations that impede market sustainability for new anti-infective drugs. Conclusions and implications of key findings: This case study highlights four pillars that may support the development of other non-traditional drugs and, concurrently, provide a new model that could reshape the field. Therapeutic triggers, study designs, and economic parameters are discussed.

## 1. Introduction

Many common infections result in substantial morbidity and mortality and represent a major strain on the healthcare system. Increasing rates of resistance to commonly utilized antibiotics only serves to exacerbate the problem [1]. Multiple companies have attempted to develop new antibiotics for use in resistant infections but have faced significant difficulty in developing novel agents that improve outcomes, address unmet needs, and, simultaneously, are economically profitable. This troubled landscape, which is littered with both scientific and commercial failures, prompts a search for new solutions.

One potential pathway for combating infection is to focus on means for looking beyond simply eradicating the culprit pathogen. These novel paradigms target virulence effectors, inflammatory responses and pathways involved in hemodynamic instability. Others even aim to bolster natural immune defenses against bacteria. These “non-traditional” therapies are not necessarily mutually exclusive, nor do they necessarily suggest pre-defined combinations, or directly amplify the activity of antibiotics or of other non-traditional drugs. Understanding how these innovative drugs overlap or differ from traditional antibiotic treatments is currently an evolving work in progress.

We present an important and unique perspective that clinicians and regulators may appreciate. Specifically, we explore the issue from a commercial angle. To this end, we discuss parameters that have defined the development of a non-traditional therapy (CAL02) that targets the essential triggers of complications—the pathogens’ virulence effectors. These critical parameters are sometimes referred to as “the value proposition” or “the critical path” of a novel therapy. First, we describe the mechanism of action and targets of CAL02 and review the key pre-clinical and clinical development issues. Second, we analyze its market strategy.

Our case study illustrates several important themes that can provide an avenue for the development of other non-traditional drugs while concurrently providing a new model that could reshape the field of anti-infective development.

## 2. Developing a Novel Anti-Virulence Drug: The Foundations

Virulence effectors form a bacterium’s key armamentarium against the host and, in turn, trigger multiple pathogenic processes. They promote bacterial colonization and growth, disrupt tissue barriers, facilitate tissue penetration and the infection invasiveness, damage tissues and organs, affect the immune balance, which further exacerbates the deterioration of the status leading to organ failure, and help bacteria wade through the innate and adaptive immune response of the host [2]. Virulence effectors, essentially, are a common denominator in severe, complicated, and resistant infections [3,4].

Anti-virulence drugs are, therefore, unique in that they address the propagation of the inflammatory response, tissue damage and organ failure—all of which are the eventual causes of death in severe infections. How do they fit into the antimicrobial field today?

First, anti-virulence drugs do not target bacteria, nor do they primarily aim at eradicating the pathogen. Rather, their potential is to prevent and treat complications associated with infection. Their action is complementary to that of antibiotics, which by their nature fail to neutralize virulence effectors. Anti-virulence drugs will not replace antibiotics. They augment them and fill a medical gap.

Second, by neutralizing upstream triggers of multiple pathogenic processes, anti-virulence drugs are expected to have a broad therapeutic impact. This contrasts with approaches targeting a downstream specific pathway causing particular inflammatory responses or organ-specific damages.

Until recently, targeting virulence seemed to be limited by specificity to a given pathogen or even a given strain. Those strategies included monoclonal antibodies targeting a single toxin, sometimes produced by a specific serotype of a given pathogen. Monoclonal antibodies have limited commercial potential given how specific they must be and, unfortunately, are complicated and expensive to develop.

**Figure 1:**
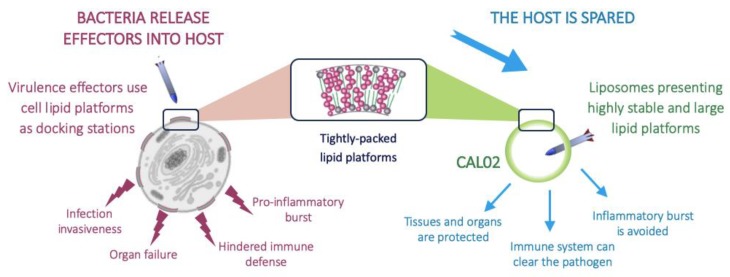
Schematic illustration of CAL02’s mechanism of action. The vast majority of virulence effectors dock on cellular lipid platforms to attack the host cells and tissues. CAL02 mimics these platforms in a highly stable manner. Virulence effectors bind to CAL02 with a higher affinity than to cells. CAL02 thus acts as a high-affinity trap.

A novel anti-virulence approach of a different nature is represented by the use liposomes (CAL02). These were specifically engineered to entrap a large variety of virulence effectors of various classes, produced by a broad spectrum of Gram-positive and Gram-negative bacteria. Rather than focusing on the identity of the toxin, this novel drug focuses on how virulence effectors attack cells. As schematized in Figure 1, this agent relies on the fact that a majority of virulence effectors target ubiquitous cell membrane lipid platforms—a highly conserved mechanism that has allowed the toxic effects of bacteria to be broad and virulence effectors to affect as many cell types as possible [5]. CAL02 mimics these lipid platforms and acts as a winning decoy efficiently sequestrating a large panel of virulence effectors, including the most frequent and relevant ones in severe infections [6]. Once trapped, virulence effectors are fully neutralized as they undergo a conformational transformation by which they significantly lose their binding and toxic capabilities [7].

Anti-virulence drugs clearly cannot be considered traditional “anti-infectives”, which are defined as medicines inhibiting the spread or killing of the infectious organism. A novel, broad-spectrum anti-virulence drug like CAL02 establishes an entirely new premise for the development of drugs to address severe and resistant infections. Pre-clinical assessments, clinical trial design, and economic concerns—all parameters used to evaluate historic anti-infectives—therefore, need to be readdressed and redefined. Choices made for CAL02’s development allowed the identification of four pillars essential for developmental success and economic sustainability.

### 2.1. Pillar 1: Target Pathogenic Triggers

**Figure 2:**
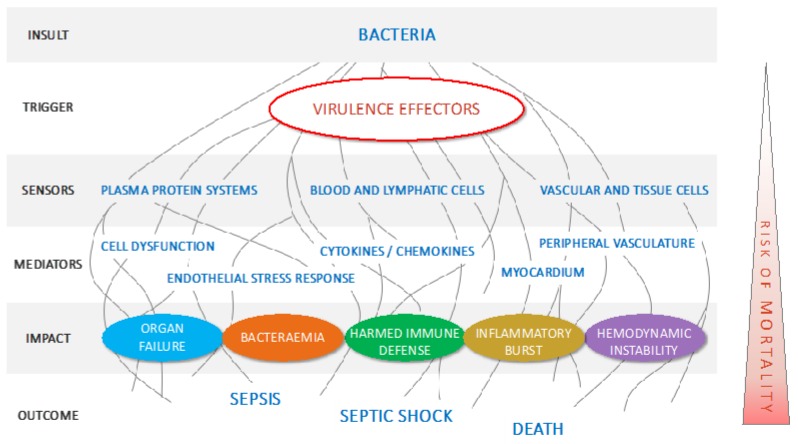
Virulence effectors as essential triggers of pathogenic pathways. Schematic illustration of the upstream role of virulence effectors in triggering multiple pathogenic processes leading to organ failure, sepsis, septic shock and death.

The first pillar is to aim at improving, significantly, treatment outcomes. To achieve this goal, targeting the triggers of pathogenic processes is key. Anti-virulence drugs target triggers of multiple pathogenic processes that antibiotics fail to neutralize (Figure 2). The tremendous impact of virulence effectors on disease severity was originally demonstrated using transgenic or knock-out mouse strains. For instance, loss of the expression of pore-forming toxins causes pathogenic bacteria to be less virulent or completely avirulent, while transgenic expression of virulence effectors in harmless bacteria turn them into aggressive pathogens [2,8]. With CAL02, the role of virulence effectors was also studied, using bacterial culture supernatant, which comprises the entire secretome of the pathogen with its panoply of virulence effectors. For instance, injection of *Staphylococcus aureus* (*S. aureus*) culture supernatant in mice caused pulmonary edema and tissue permeability, and resulted in the death of all animals within 30 h. In the absence of direct bacterial challenge, severe and fatal organ damage was similar to that caused by infection and was characteristic of severe infections caused by *S. aureus*. Pre-incubation of the secretome with CAL02 fully abolished the pathogenic impact and all animals survived [6].

The ability to act on virulence rather than on a single bacterial weapon is another component of this pillar. As mentioned above, so far, anti-virulence strategies have mainly consisted of narrowly focused monoclonal approaches.

Virulence effectors produced by a given pathogen are numerous. Moreover, they may be produced in various amounts by different strains, and they may act at different moments throughout the course of the infection. A drug capable of neutralizing a broad panel of virulence effectors is thus expected to exert a stronger therapeutic effect. This was examined in vivo using CAL02 as the sole therapeutic intervention. Indeed, while monotherapy studies (no antibiotics) are not strictly supportive of the primary clinical use of an anti-virulence drug, which will be administered alongside antibiotics, they reveal the importance of the therapeutic targets. Several animal models mimicking human infections were used: pneumonia caused by *Streptococcus pneumoniae* (*S. pneumoniae*) and *Pseudomonas aeruginosa* (*P. aeruginosa*), bacteremia caused by *S. pneumoniae* and *S. aureus*, and skin infection caused by the methicillin-resistant *S. aureus* (MRSA) USA300 strain [6,9,10]. CAL02 on its own was able to provide full protection (100% survival) and its protective impact on organ damage was significant. For example, in a systemic acute infection model caused by *S. aureus* causing 100% death in approximately 48 h, CAL02, administered 2, 6, and 24 h after bacterial challenge at doses corresponding to those used in the clinic, was shown to provide full protection. There was a 100% survival rate and lungs remained intact (no lung edema as indicated by comparing wet/dry weight of the lungs and by monitoring the intrapulmonal leakage of Evans Blue) [6]. These studies also highlighted the impact of the intervention on pro-inflammatory responses associated with the infection (e.g., impact on cytokine levels including tumor necrosis factor (TNF)-α and interleukin (IL)-1beta, reduction of the pro-inflammatory recruitment of blood polymorphonuclear leukocytes) and on protecting the integrity of the first line of immune defense (e.g., attenuation of bacteremia-induced reduction of blood B-cells, protection of leukocytes attacked by bacteria). In fact, CAL02 neutralizes *S. aureus*’ α-toxin as well as the pore-forming leukotoxin, Panton–Valentine leukocidin (PVL) and other leukocidins, gamma (γ)-Haemolysins A and B (HlgA, HlgB), hemolysin-β (sphingomyelinase C), and phenol-soluble modulins (PSMs) [9]. All these virulence effectors play an essential role in the development of complications and target lipid platforms at the surface of host’s cells (those platforms mimicked by CAL02) to exert extensive toxic effects on the host [11].

Monotherapy studies with CAL02 and other anti-virulence drugs have also shown that although the drug has no bactericidal activity, it deprives bacteria of mechanisms used to feed and multiply and it acts as a shield for the immune system, which can then clear the infection more appropriately [6,12,13]. These results further underline the relevance of these therapeutic targets and the potential of these new drugs against multi-drug-resistant strains. They also suggest the potential to improve current practice by providing the much-needed time that allows antibiotics to realize their full anti-bacterial activity.

### 2.2. Pillar 2: Timely Intervention

To illustrate this second pillar, we examined a major medical and economic problem worldwide: pneumonia. Pneumonia represents the second most frequent cause of hospitalization and is ranked among the leading cause of death worldwide [14,15]. Pneumonia further represents the most common infection requiring admission to the intensive care unit (ICU). Strikingly, acute, long-term, and even lethal complications most often occur when tissues are already pathogen-free and the pulmonary process is clearing [16]. These complications are multiple: bacteremia, sepsis, pleural effusion, empyema, respiratory failure, abscesses, acute coronary syndromes, endocarditis, heart failure, acute kidney failure, cognitive deficits, and nosocomial co-infections.

Historically, antibiotics have been the weapon of choice. However, despite best available treatments and new antibiotics, complications have not decreased, and mortality rates associated with pneumonia and its complications range from 35% to 58% [17,18,19,20]. New strategies and novel approaches are imperative. Irrespective of whether the culprit pathogen is resistant or sensitive to antibiotics, bacterial strains involved in severe pneumonia employ virulence effectors causing complex systemic inflammatory response and widespread damage [15].

Current treatments for severe pneumonia mainly reside in antibiotic therapies [19,21]. Beyond antibiotics, treatment and management strategies more specific to patients developing sepsis or septic shock involve fluid therapy and vasoactive medications, corticosteroids, mechanical ventilation, renal replacement therapy, venous thromboembolism prophylaxis, and stress ulcer prophylaxis [22]. Other strategies in development encompass approaches or drugs focusing on hemodynamic stability and shock. Some novel drugs target very specific host components involved in immune imbalance, cytokine storm and specific organ function. These strategies are generally used when severity is well engaged and/or patients are already in shock.

Like other non-traditional agents, anti-virulence drugs exhibit no bactericidal activity. The usual MIC (minimum inhibitory concentration) and MBC (minimal bactericidal concentration) breakpoints applied for antibiotic efficacy assessment are not applicable in this case [23]. New standards are thus required. Pre-clinical work with CAL02 focused on assessing the impact of the treatment on survival, on organ function, and on inflammatory response, in line with the mechanism of action of the drug.

**Figure 3:**
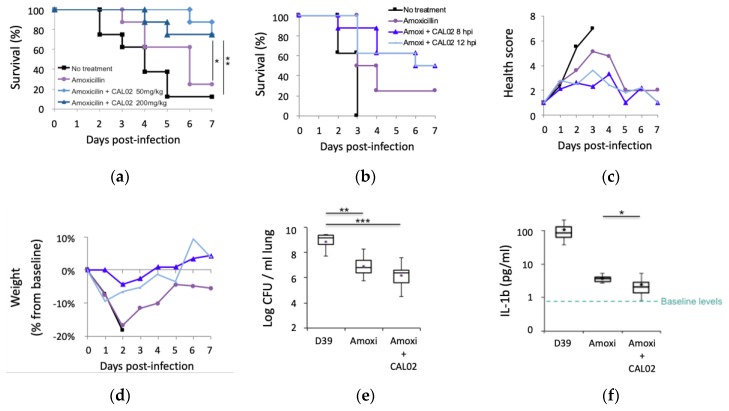
CAL02 in addition to antibiotics in acute pneumonia caused by *S. pneumoniae*. CD-1 mice were challenged with a lethal intranasal infection of *S. pneumoniae* D39. A single dose of amoxicillin (0.2 mg/kg in (**a**–**d**), 1 mg/kg in (**e,f**)) was administered subcutaneously at 4 h post-infection. Study (**a**): A single dose of CAL02 (50 or 200 mg/kg) was administered intravenously at 4 h post-infection. Study (**b**): A single dose of CAL02 (50 mg/kg) was administered intravenously at 8 or 12 h post-infection. At the end of the study, all surviving mice treated with CAL02 had fully recovered as indicated by health scores(**c**) and weight (**d**). (**e,f**) Impact of CAL02 (200 mg/kg) administered 6 h after antibiotics on bacterial loads in lungs (**e**) and on blood IL-1beta (**f**), measured at 30 h post-infection; (**a**–**e**) *n* = 8 per group; (**f**) *n* = 4 in the untreated group; and *n* = 8 in treated groups. * *p* < 0.05, ** *p* < 0.01, *** *p* < 0.005; Log-rank (Mantel–Cox) test *p* < 0.05. [25].

In vitro experiments demonstrated the positive impact of CAL02 on cell protection (protection against cell lysis and cell necrosis) and on inflammatory response (e.g., reduction of IL-8 and IL-1beta release), using cell lines as broad as human THP-1 monocytes, human peripheral blood mononuclear cells, bronchial and pharyngeal epithelial cells, HEK 293 epithelial cells, human umbilical vein endothelial cells (HUVEC), and erythrocytes. Cells were exposed to purified bacterial virulence effectors of different classes or to bacterial culture supernatant (i.e., to the secretome, which contains the full range of virulence effectors secreted by the bacterium), or directly exposed to the bacteria [6,9,10,24]. In vivo studies included acute models of infection caused by Gram-positive (*S. aureus* and *S. pneumoniae*) and Gram-negative (*P. aeruginosa*) bacteria, including resistant strains [6,9,10,25,26]. As illustrated in the studies depicted in Figure 3, particular attention was devoted to simulating clinical settings: animals treated hours after the infectious challenge and CAL02 administered in addition to antibiotics, or even hours after antibiotics [25]. Notably, in an acute pneumonia model caused by *S. pneumoniae*, a single administration of CAL02 given simultaneously with antibiotics 4 h post-infection (Figure 3a) or given 8 or 12 h post-infection and, thereby, 4 or 8 h after antibiotics (Figure 3b), provided significantly higher protection than antibiotics alone. At the end of the studies, all surviving mice treated with CAL02 had fully recovered as indicated by health scores and weight (Figure 3c,d). These studies also evaluated the impact of the treatment on bacterial loads (Figure 3e) and on inflammatory responses. For instance, CAL02 administered 10 h post-infection (6 h after antibiotics) led to a greater decrease in levels of IL-1beta, which is an important early mediator of pro-inflammation, which contributes to up-regulation of other pro-inflammatory mediators, and which persistently increases in fatal sepsis (Figure 3f).

Patients with severe pneumonia would potentially greatly benefit from a treatment that operates on both fronts: killing bacteria and neutralizing virulence effectors. For such a treatment to succeed, however, it should meet the constraints of the clinical scenario and be able to be given rapidly and before knowing the specific causative pathogen. Furthermore, numerous recent studies have underlined the fact that rapid intervention is critical in pneumonia [17,21,27,28,29]. For severe community- and hospital-acquired pneumonia, considering strain frequency on the one hand and severity and mortality on the other hand, the most relevant pathogens are *S. pneumoniae, S. aureus* and *P. aeruginosa* [15,27,29,30,31], which explains the focus in studies with CAL02 given what has been demonstrated in pre-clinical evaluation.

The drug could also be one clinicians would use without significant safety concerns. Moreover, as it is not an antibiotic, it would not promote further resistance. A drug targeting virulence effectors imposes no selective pressure on intrinsic bacterial survival pathways. It acts on effectors produced by bacteria; chances of feedback that toxins released by the pathogen have been trapped are low.

### 2.3. Pillar 3: Target for Comprehensive and Hard Efficacy Endpoints

In clinical development, the traditional endpoints for trials focus on clinical cure and mortality. That being said, recent trials in severe pneumonia underscore that, in the majority of the cases, patients who are considered “cured” continue to suffer from acute and long-term complications. These complications are responsible for the economic burden of the disease and for much of the mortality [18,19,20,32,33,34]. Bacteria have triggered pathogenic processes against which antibiotics, themselves, are powerless. This was also observed in the first in-human clinical study with CAL02. Although the standard antibiotic therapy was able to achieve a 100% cure rate within 5–10 days, there were important differences in the time to normalization of organ functions and mortality between patients given only antibiotics and those given antibiotics and CAL02 [35].

For drugs that are not intended to treat the infection per se, but rather the damaging consequences of the infection, the definition of “cure” is inadequate. Efficacy should be appraised using parameters that reflect the agent’s mechanism of action. A significant improvement in treatment outcome means saving patients, but also means successfully treating those who survive—still the majority—and are suffering from complications. For severe pneumonia this could be captured by focusing on organ failures, progression to sepsis, and development of respiratory failure necessitating mechanical ventilation.

In the first-in-human clinical study with CAL02, numerous assessments of the clinical efficacy and pharmacodynamic characteristics were performed [35,36] They included clinical cure as well as mortality. On Day 8, 56% of the CAL02-treated patients were cured from the pneumonia episode, versus 20% in the placebo arm. Survival was also favored in the treatment arm, with a rate of 10%, despite an approximated mortality risk of 40% at baseline based on the Acute Physiology and Chronic Health Evaluation (APACHE) II) score, a mortality predictor. Moreover, between baseline and Day 8, the APACHE II score decreased by a mean of 60% in the CAL02 groups compared with 22% in the placebo group, which further points to the impact of the treatment on survival outcome. These data serve as proof of concept that anti-virulence strategies augment antibiotics and help patients to recover more quickly.

Of course these initial clinical findings with CAL02 are preliminary and derive from a small sample size. Hence, they must be viewed with caution. However, as underlined by Pletz and colleagues, the treatment advantages observed over current treatments were reinforced by the fact that they were consistently observed across all efficacy assessments—mortality and early cure, as well as scores indicating patients’ status and severity, inflammation biomarkers, days under vasopressors and days under invasive mechanical ventilation, duration of stay in the ICU, etc. [37].

Among these assessments, one should particularly underline those that correlate with the mechanism of action of CAL02, and at the same time correspond to the expected treatment impact, can be measured objectively (are “hard” endpoints), and have been extensively tested and validated in medical practice. For example, the Sequential Organ Failure Assessment (SOFA) score is a well-established and validated tool commonly used to assess the status of severe pneumonia patients in the ICU. It indicates a sequence of complications and is based on six different scores related to respiratory, cardiovascular, hepatic, coagulation, renal and neurological systems. This score is also the selected tool to assess evidence of organ failure according to the very recent third international consensus definitions for sepsis and septic shock: Sepsis-3 [38]. A higher SOFA score is associated with an increased probability of mortality [39]. In the first in-human clinical study with CAL02, the SOFA score between baseline and Day 8 decreased by a mean of 65% in the CAL02 arms versus 29% in the placebo arm [35]. This treatment effect was statistically significant (*p* = 0.022). The cardiovascular SOFA subscore of the CAL02 arm also showed a complete normalization by Day 6, contrasting with persistent instability in the placebo arm. Composite endpoints comprising early assessments of recovery such as the SOFA score have been selected as primary endpoints in numerous recent, ongoing, and soon to-be-initiated Phase 2 and 3 clinical studies carried out in patients with severe infections, including pneumonia and sepsis (Table 1). The evolution of organ failure correlates with the expected protective and therapeutic impact of CAL02.

### 2.4. Pillar 4: Aiming at Economic Sustainability

Beyond all the differences noted above between traditional antibiotic and anti-virulence approaches, the economic issues surrounding the development of these agents also are distinct.

First, while a vast majority of antibiotics have been approved based on non-inferiority clinical trial designs, following EMA and FDA guidelines [40,41], adjunctive therapies that target virulence factors afford the chance to finally perform superiority studies in the field of severe infection. Unlike non-inferiority studies, which often leave clinicians wondering how to employ a novel therapy, a superiority clinical trial—akin to designs seen in cancer clinical studies—makes it easier for physicians to determine how to utilize and adopt a newly approved treatment. This potentially leads to faster clinical adoption and commercial success.

Second, in contrast to new antibiotics, which, in most cases, have been restricted to a limited market designed for patients with no alternative treatment options, anti-virulence drugs address the severe consequences of infections affecting millions of individuals every year. They do not compete with current treatments. On the contrary, they complement antibiotics, which will be, in any case, administered to patients as a direct attack on bacterial survival. Their market is real and broad-based, which reinforces the potential for commercial success and, in turn, can draw financial support back into this neglected space.

Third, these non-traditional drugs can aspire to rapid and wide market adoption, not only because they aim at superiority of outcome, as noted above, but also because they can effect measurable and meaningful outcomes. Infectious complications result in significant increases in rates of hospitalization and lengthy inpatient care, which place a considerable burden on healthcare resources. Inpatient care accounts for approximately 80% to 95% of the total costs associated with community-acquired pneumonia, and re-admission rates can reach 20% within the first 30 days of disease onset [42,43,44,45]. The direct annual cost of community-acquired pneumonia is estimated to be approximately €10 billion in Europe and $17 billion in the United States [42,45,46]. Complications in hospital-acquired pneumonia increase the duration of hospital stay by more than a week, increase costs by up to €40,000, and result in a threefold increase in mortality [28]. These facts and numbers are further exacerbated by the increasing rate of occurrence of infections caused by strains resistant to antibiotics [1]. Efficacy outcomes examined in the first in-human clinical study with CAL02 also included outcomes related to health economics [35]. CAL02-treated patients recovered faster, required a shorter period under invasive mechanical ventilation (4.5 days in the CAL02 arm versus 12 days in the placebo arm) and stayed in the ICU for a significantly shorter period (5 days (range 2–15) in the CAL02 arm versus 12 days (range 6–56) in the placebo arm (*p* = 0.027, log-rank (Mantel–Cox) test)).

## 3. Conclusions and Implications of Key Findings

Eliminating bacteria is of paramount importance but is clearly not enough. Bacteria, whether sensitive or resistant to antibiotics, stimulate sensors and trigger damaging pathways (see Figure 2). In the severe and resistant infection arena, there is largely room for improvement in the full and rapid restoration of health.

The critical pillars exposed in this review outline how a non-traditional anti-virulence drug aims at filling this medical gap. This type of non-traditional drug targets essential triggers of pathogenicity, and its characteristics allow timely administration when hours count in the prevention of clinical status deterioration.

Non-traditional drugs have the potential to address medical needs affecting millions of individuals every year. Importantly, these new drugs do not compete with current treatments and do not aim to replace antibiotics. They are not “alternatives”. Rather, they aim to provide a significant added value to existing treatments, without inflicting selective pressure and without damaging the microbiome. Furthermore, economic sustainability is likely, based on scientifically rational principles supportive of commercial investment in a novel paradigm for treating severe infections. They may, in fact, enable those companies developing antibiotics who desire to, additionally, develop non-traditional drugs to contemplate commercial success.

Although companies developing non-traditional drugs may not specifically develop target product profiles focused on specific resistant strains and niche indications, non-antibiotic drugs in their own right contribute to the fight against resistance. In fact, anti-virulence drugs do not add to the resistance burden and are active irrespective of antimicrobial resistance. Because anti-virulence drugs neutralize effectors active in bacterial expansion, and act as a shield for the immune system, which can clear infection more efficiently, they have the potential to combat multi-drug resistant infections. Moreover, they act on bacterial effectors that participate in the expansion and consolidation of resistance mechanisms that favor opportunistic infections by resistant strains. They clearly contribute, therefore, to long-term solutions against the spread of resistance.

A change of paradigm is necessary and is already happening. Bacterial multiplication is not the sole enemy in the battle against bacterial infections. The real therapeutic challenge is to treat the damage infections wreak on the body. Not relying solely on MIC involves taking into account the clinical reality. Aiming at superiority means working for progress.

## Figures and Tables

**Table 1:** Examples of recent or current clinical studies in severely infected patients.

Clinical Study National Clinical Trial (NCT) Identifier Phase—Status	Indication Study Drug	Primary Efficacy Endpoint(s)
**Antibiotics**		
Comparison of Two Antibiotic Regimens in the Treatment of Severe Sepsis and Septic Shock (MaxSep) NCT00534287Phase 3—Completed (2010)	Severe sepsis/Septic shock Meropenem vs. Meropenem plus Moxifloxacin	Mean total SOFA score (study duration or up to Day 14)
Clinical Outcome Study of High-Dose Meropenem in Sepsis and Septic Shock PatientsNCT03344627 N/A—Completed (2018)	Sepsis/Septic shock Meropenem	Change of total SOFA score from Baseline to Day 4
**Immuno-Modulator Approaches**		
Cx611-0204 SEPCELL Study (SEPCELL) NCT03158727 Phase 1/2—ONGOING	Severe CABP Cx611 (allogeneic adipose-derived stem cells)	Composite: Reduction of the duration of mechanical ventilation and/or vasopressors needed and/or improved survival, and/or clinical cure of the CABP, and other infection-related endpoints
Esomeprazole to Reduce Organ Failure in Sepsis (PPI-SEPSIS) NCT03452865 Phase 3—Not Yet Recruiting	Sepsis/Septic shock Esomeprazole	SOFA score reduction (Days 1–28)
Efficacy, Safety and Tolerability of Nangibotide in Patients with Septic Shock (ASTONISH) NCT04055909 Phase 2—ONGOING	Septic shock Nangibotide (formerly LR12, TREM-1 inhibitor)	Change of total SOFA score from baseline to Day 3 (in the subgroup defined by patients with elevated sTREM-1 baseline levels and in the overall population)
**Approaches Targeting Hemodynamic Instability and Shock**		
Selepressin Evaluation Programme for Sepsis-Induced Shock—Adaptive Clinical Trial (SEPSIS-ACT) NCT02508649Phase 2/3—Completed (2018)	Septic shock Selepressin	Vasopressor- and mechanical ventilator-free days: Defined as number of days from start of treatment to 30 days thereafter during which the patient is 1) alive; 2) free of treatment with vasopressors; 3) free of any mechanical ventilation
Rapid Administration of Carnitine in sEpsis (RACE) NCT01665092 Phase 2—Completed (2019)	Septic shock Levo-Carnitine	Delta SOFA Score (48 h)
Treatment of Patients with Early Septic Shock and Bio- ADM Concentration > 70 pg/mL With ADRECIZUMAB (AdrenOSS-2) NCT03085758 Phase 2—ONGOING	Septic shock & ADM > 70 pg/mL ADRECIZUMAB (monoclonal antibody targeting adrenomedullin)	SSI within 14 day follow-up defined as follows: Each day on vasopressor, and/or mechanical ventilation, and/or renal failure (defined as renal SOFA = 4), or not alive, is counted 1; the sum over the follow up period is defined as SSI. Among secondary outcomes: SOFA score and its changes over time (composite)
Remote Ischemic Conditioning in Septic Shock (RECO-Sepsis) NCT03201575 N/A—ONGOING	Septic shock Remote ischemic conditioning (inflations and deflations of a brachial cuff)	Average SOFA score (96 h)
Efficacy and Safety of Rheosorbilact^®^ Solution for Infusion, in a Complex Therapy of Pneumonia NCT03824457 Phase 4—ONGOING	CAP with PSI/PORT index score ≥ IV and SOFA ≥ 2 points and < 48 h since beginning of antibacterial therapy Rheosorbilact^®^	A change in the total SOFA score (while at ICU) vs. baseline score upon admission
Efficacy and Safety of Rheosorbilact^®^ Solution for Infusion, in a Complex Therapy of Sepsis NCT03764085 Phase 4—Completed (2020)	Sepsis Rheosorbilact^®^	A change in the total SOFA score (while at ICU) vs. baseline score upon admission
Ilomedin in Septic Shock with Persistent Microperfusion Defects (I-MICRO) (I-MICRO) NCT03788837 Phase 3—Not yet recruiting	Septic Shock Hyperdynamic Ilomedin (prostaglandin analog)	Delta SOFA score between infusion onset and Day 7 and patients deceased before Day 7 will be attributed a maximum SOFA score.
Guided Fluid-Balance Optimization with Mini-Fluid Challenge During Septic Shock (GOAL) NCT03461900 N/A—Not Yet Recruiting	Septic shock Minifluid challenge	Delta SOFA score (between Day 0 and 5)
**Hemoadsorbers**		
Adsorbtion of Cytokines Early in Septic Shock: The ACESS Study NCT02288975 Medical device—Completed (2017)	Septic shock CytoSorb 300 mL device (3804606CE01)	Cytokine response AND Organ dysfunctions (incl. SOFA) In the first 48 h of septic shock
A Double-Blind, Randomized Placebo-Controlled Clinical Investigation With Alteco^®^ LPS Adsorber (ASSET) NCT02335723 Medical device—Completed (2017)	Septic shock Alteco^®^ LPS Adsorber	Relative change from baseline in SOFA score (6–28 days)
Hemoadsorption for Prevention of Vasodilatory Shock in Cardiac Surgery Patients with Infective Endocarditis (REMOVE) NCT03266302 Medical device—ONGOING	Infective Endocarditis Hemoadsorber for removal of cytokines	Mean SOFA score (between 24 h before until day 9 post-surgery)
Use of Extracorporeal Treatment with the Cytosorb-Adsorber for the Reduction of SIRS in Heart Surgery Patients (CASHSP) NCT02265419 Medical device—ONGOING	Heart surgery with SIRS criterions and postoperative central venous oxygen saturation >75% and need of vasopressors within 6 h postoperative Extracorporeal treatment with the Cytosorb adsorber	Mean SOFA score (to Day 7)

Abbreviations: ADM: adrenomedullin; CABP: community-acquired bacterial pneumonia; CAP: community-acquired pneumonia N/A: not applicable; PORT: pneumonia patient outcomes research team; PSI: pneumonia severity index; SIRS: systemic inflammatory response syndrome; SOFA: sequential organ failure assessment; SSI: sepsis support index.
